# Drift-dependent changes in iceberg size-frequency distributions

**DOI:** 10.1038/s41598-017-14863-2

**Published:** 2017-11-22

**Authors:** James D. Kirkham, Nick J. Rosser, John Wainwright, Emma C. Vann Jones, Stuart A. Dunning, Victoria S. Lane, David E. Hawthorn, Mateusz C. Strzelecki, Witold Szczuciński

**Affiliations:** 1 Geography Department and Institute of Hazard Risk and Resilience, Durham University, Durham, DH1 3LE UK; 2 Scott Polar Research Institute, University of Cambridge, Cambridge, CB2 1ER UK; 3 School of Geography, Politics and Sociology, Newcastle University, Newcastle, NE1 7RU UK; 4 SEIS-UK, Department of Geology, University of Leicester, Leicester, LE1 7RH UK; 5 British Geological Survey, The Lyell Centre, Edinburgh, EH14 4AP UK; 6 Institute of Geography and Regional Development, University of Wrocław, 50-137 Wrocław, Poland; 7 Institute of Geology, Adam Mickiewicz University in Poznań, 61-680 Poznań, Poland

## Abstract

Although the size-frequency distributions of icebergs can provide insight into how they disintegrate, our understanding of this process is incomplete. Fundamentally, there is a discrepancy between iceberg power-law size-frequency distributions observed at glacial calving fronts and lognormal size-frequency distributions observed globally within open waters that remains unexplained. Here we use passive seismic monitoring to examine mechanisms of iceberg disintegration as a function of drift. Our results indicate that the shift in the size-frequency distribution of iceberg sizes observed is a product of fracture-driven iceberg disintegration and dimensional reductions through melting. We suggest that changes in the characteristic size-frequency scaling of icebergs can be explained by the emergence of a dominant set of driving processes of iceberg degradation towards the open ocean. Consequently, the size-frequency distribution required to model iceberg distributions accurately must vary according to distance from the calving front.

## Introduction

The rate at which icebergs drift and disintegrate influences the risk of collisions with high-latitude hydrocarbon infrastructure and shipping^1^, the extent of zones of nutrient-enhanced carbon sequestration^2,3^, and the interpretation of palaeoclimate indicators such as ice-rafted debris^4^. Although iceberg drift-decay models exist^5^, our mechanical understanding of iceberg disintegration remains unable to explain the size-frequency distributions of icebergs commonly observed; most notably the discrepancy between the power-law distributed icebergs sizes observed at glacial calving fronts^6^ and the lognormal iceberg-size distributions observed globally within open waters^7,8^. Although it has been speculated that the lognormal distribution of iceberg sizes observed away from glacial calving fronts is the product of the mechanisms by which icebergs fracture and disintegrate^7^, the absence of appropriate methods with which to study free-floating iceberg disintegrations has limited efforts to study the mechanics of this phenomenon.

Over the last four decades^9^, passive seismic investigations of glaciological phenomena have revealed that different glaciological processes are characterised by unique and highly distinctive signal properties including dominant spectral frequency, event duration and the shape of the signal onset and coda^10^ (Table 1). The application of passive seismic techniques has significantly increased our understanding of inaccessible glaciological processes including crevasse propagation^9,11,12^, basal sliding^13,14^ and iceberg calving from tidewater glaciers^15,16^. Seismic methods have also been used to describe flexure and breakage of free-floating tabular icebergs^14,17^, demonstrating their potential to provide insight into the mechanisms responsible for iceberg disintegration.

**Table 1:** Glaciological processes known to generate seismic tremors and their associated waveform geometry, frequency and duration. Note that iceberg calving, grounding and ice mélange interaction processes are associated with a significantly longer duration (in the order of minutes to hours) than the other phenomena which may be measured on timescales in the order of seconds.

Glaciological process	Waveform geometry	Characteristic frequency (Hz)	Typical duration	References
Surface crevassing and ice fracture	Impulsive onset and abruptly declining coda	10–30	0.1–2.5 (s)	7,9,12,30
Iceberg calving and capsize	Emergent onset, cigar-shaped envelope, long-duration coda, absence of P- or S-waves, peaks often coincide with ‘Worthington jets’ produced by cavity collapse	1–5	5–30 + (s) (up to 1 hour depending on iceberg dimensions)	7,37,38,64
Basal sliding	No surface waves	1–25	—	14
Iceberg interaction with ice mélange	Multiple harmonic frequencies	0.5–30 with multiple harmonics	30–60 (minutes)	37
Hydraulic movement in glacial water channels	Emergent onset, lack of distinct S-waves	6–15	1–10 (s)	10
Iceberg grounding and ploughing	Long duration, monochromatic frequency	0.5–1.5	~2 (hours)	14
Hydrofracturing	Impulsive onset	20–35	1–10 (s)	10
Iceberg harmonic tremor	Multiple harmonic frequencies with a distinctive ‘chevron’ pattern	1–10 with multiple harmonics	1500 (s)	65

The Greenland Ice Sheet has experienced persistent and increasing mass loss since the 1990s^18^ in a spatially complex pattern driven by rising surface air temperatures^19^ and accelerations in outlet glacier velocities^20,21^. During this time, freshwater fluxes into the North Atlantic Ocean sourced from surface and submarine melting of the Greenland Ice Sheet, as well as the melting of icebergs and ice mélange, have been observed to increase^22,23^. In addition to their implications for circulation dynamics within the global ocean^24^ and mass-loss feedbacks within the fjords of marine-terminating outlet glaciers^25^, elevated meltwater fluxes are likely to increase the input of bioavailable particulate iron into the North Atlantic Ocean^3^, potentially affecting marine biological productivity, ecosystem dynamics and the oceanic uptake of CO_2_^2^. Meltwater fluxes sourced from the melting of icebergs and ice mélange within Greenlandic glacial fjords including that of Greenland’s large outlet glacier, Jakobshavn Isbræ^26^, may potentially exceed the flux associated with glacier surface and submarine melting^22^. The drift and decay of icebergs during transit from the calving terminus therefore represents an important mechanism by which nutrients and freshwater are transported into the North Atlantic Ocean; however, due to a poor understanding of iceberg-disintegration mechanics, these processes are relatively poorly quantified around Greenland at present^3^.

**Figure 1:**
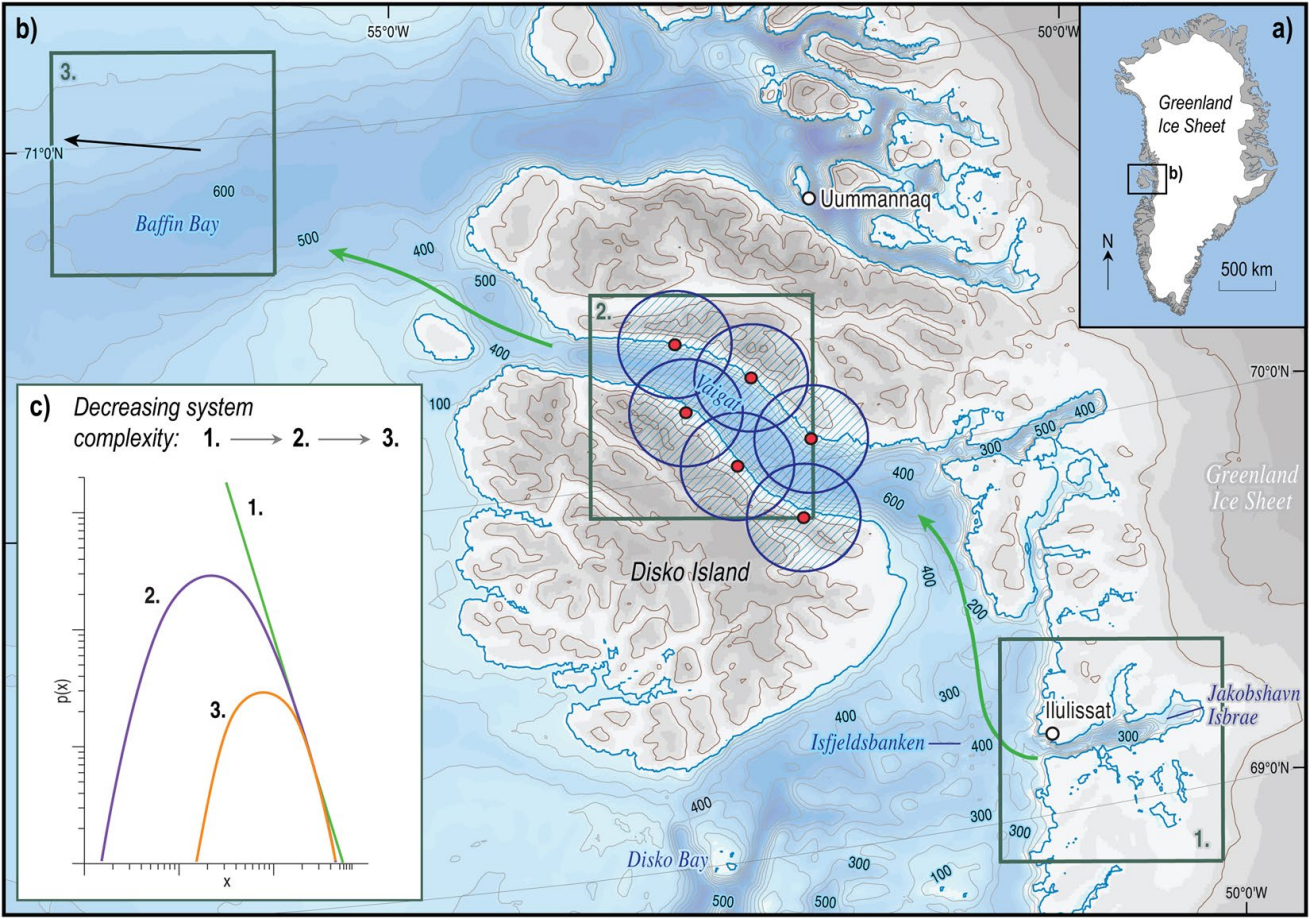
The lognormal shift in iceberg size magnitude-frequency distributions with distance away from the calving front at Jakobshavn Isbræ. (**a**) Location of the study site in West Greenland and (**b**) the area surrounding Vaigat displaying regional bathymetry and the locations referred to in the text. Seismometers are displayed as red dots with their 15 km effective detection radiuses shown as blue circles. (**c**) Idealised comparison between the probability distributions, p(x), of an inverse power-law (green line 1) and two lognormal distributions (curves) of decreasing complexity, labelled 2. and 3. As the hierarchy of processes responsible for the lognormal distribution becomes more complex (curve 3. to 2), the distribution becomes broader, providing a greater degree of overlap with the inverse power-law distribution^53^ (line 1). The complexity of the breakage process responsible for generating the magnitude-frequency distribution of iceberg sizes decreases with distance away from the calving front of Jakobshavn Isbræ owing to the emergence of a dominant set of decay mechanisms. As a result, the power-law distribution of iceberg sizes initially present proximal to Jakobshavn Isbræ (1) evolves towards lognormal scaling, and becomes more characteristically lognormal, as icebergs transit from Jakobshavn Isbræ to Vaigat (2) and towards the open ocean (3). Bathymetric data is obtained from the IBCAO V. 3.0 dataset^63^ and drawn using ArcMap 10.3.

If an accurate understanding of the mechanisms of iceberg breakup could be obtained, numerical models could be used to predict the expected distribution of iceberg sizes resulting from the disintegration process, their trajectories, and their longevity; information which informs risk to shipping and delineates the areas influenced by the delivery of ice-rafted debris and nutrients. One such modelling approach is the use of probabilistic magnitude-frequency scaling laws, which provide a means to quantify the likelihood that an event of a given magnitude will occur over time or, in the context of icebergs, that an iceberg of known dimensions will be produced as a result of the disintegration process. This approach has been widely applied in attempts to forecast the occurrence of natural hazards such as rockfalls^27^ and landslides^28^. We therefore apply a probabilistic scaling approach to capture, characterise and model the manner in which icebergs calved from Jakobshavn Isbræ disintegrate as they drift through the Vaigat Strait towards Baffin Bay, West Greenland, determined through passive seismic monitoring (Fig. 1). Based on a lognormal distribution of energy released by iceberg cracking and calving, we conclude that the lognormality associated with free-floating iceberg size-frequency distributions is a product of the process of iceberg disintegration and dimensional reductions through melting after their initial calving. We propose that the emergence of a dominant set of iceberg-degradation processes over space transforms the characteristic distribution of iceberg dimensions from a power-law at glacial calving fronts to a lognormal distribution as icebergs drift towards the open ocean.

## Results

### Description and interpretation of seismic events

Seismic signals generated by the processes of iceberg decay were recorded over a 49-day period using a network of six seismometers installed in coastal locations along a 50 km stretch of the Vaigat Strait. Based on their distinctive characteristic spectral frequencies, event durations and signal onset and coda geometries, the observed iceberg-related seismic events may be classified into three groupings, implying that three predominant processes are responsible for generating seismicity in the Vaigat Strait. The waveform geometry, typical duration and characteristic frequencies of the three classes of signals compare favourably to previously examined glaciological processes (Table 1), suggesting that the seismic signatures of iceberg decay observed within the Vaigat Strait relate to cracking, microfracturing and iceberg-calving processes (Table 2) (Supplementary Figs 1 and 2).

**Table 2:** Characteristic frequencies, duration and waveform descriptions for the three types of events detected by the seismometer array over a 49-day period.

Signal type	Description of signal onset	Description of signal coda	Characteristic frequency (Hz)	Typical duration (s)	Number detected	Process interpretation
1	Impulsive	Abrupt termination	30–40	1	1979	Microfracturing
2	Impulsive	Abrupt termination	10–30	1–5	3271	Tensile fracturing and crack enlargement
3	Emergent; amplitude increases over time	Gradual decline in amplitude	1–10	3–15	1592	Iceberg calving, capsize and rolling

The first class of icequake, Type 1, exhibits an impulsive, short duration (~1 s) waveform with a characteristic frequency of 30–40 Hz. The brittle nature of ice means that mechanisms of ice deformation are dominated by fracturing, resulting in micro-cracking, coalescence of fractures and fragmentation as the ice exceeds a critical threshold of viscoelastic strain^29^. Ice crevassing and surface fracture is typically associated with short duration (~0.1–2.5 s), 10–30 Hz seismic tremors with highly impulsive onsets^9,30^. These properties are consistent with the characteristics of Type 2 icequakes, implying that this signal type likely corresponds to tensile fracturing and the enlargement of pre-existing cracks and crevasses. Depending on the mode of failure, fractures may open through tension-based or shearing-dominated mechanisms^31^. These two mechanisms may be differentiated by inspecting the polarity of the first motion of the seismic signal — with consistent first motion polarity across all sensors indicative of tensile failure and mixed polarity signifying that the source has some shearing component^32,33^. Although it is often difficult to distinguish the onset of a signal from the pre-event noise, Type 2 signals generally exhibit a consistent polarity of first tremor motion, supporting the interpretation of this signal as originating from the tensile failure of ice.

The similar waveform geometry, duration and first motion polarity of Type 1 and Type 2 icequakes suggests that these signals share a similar genesis. The characteristic frequency of seismic waves resulting from brittle material failure scales in accordance with the size of the fracture and the shear modulus of the medium^10,12^. The basic response frequency for fractures in ice, *f* [Hz], has been shown to respond to changes in crack length^34,35^, *L* [m]:

1 $$f=\frac{V}{2L}$$

where *V* is the typical crack propagation velocity for ice [m s^−1^]. Laboratory and large-scale geophysical experiments have demonstrated that the mean velocity of a simple crack in ice is approximately 50 m s^−1^ and thus, assuming a constant shear modulus, smaller length cracks will result in a higher characteristic frequency relative to larger crack lengths^35,36^. On the basis of this relationship, Type 2 signals correspond to 0.8–2.5 m crack lengths whereas Type 1 signals relate to smaller microfractures with lengths less than 0.8 m.

Type 1 signals are frequently detected prior to and after the onset of Type 2 signals, suggesting that these types of events may be mechanically linked. Cracking and micro-fracturing are progressive processes in which fractures radiate outwards from the tips of cracks following the exceedance of interatomic bonding forces by local tensile stresses^35^. Consequently, the co-occurrence of these two signals likely reflects micro-fracture nucleation at the tips of an enlarging crack, instigated by the volumetric enlargement of the pre-existing rupture. Hence, signal types 1 and 2 appear to be part of a genetically related continuum of tensile fracture processes.

The emergent onset, gradually declining coda and predominantly monochromatic 1–5 Hz spectral frequency of the Type 3 signals is consistent with the characteristics of iceberg calving from glacial termini^7,37^. The low frequency (1–5 Hz) spectral peak associated with this type of event has been attributed in previous studies to iceberg-water interactions through both displacement of water following iceberg collisions with the water surface^38^ and the tilting and rolling of unstable icebergs following detachment from the calving terminus^39^. Whilst the dominant spectral frequency of Type 3 events corresponds to the 1–5 Hz frequency band, this type of signal commonly contains a number of short-lived 20–40 Hz peaks, similar to the Type 1 and 2 icequakes, prior to and within the main body of the signal. Type 3 events may therefore consist of the tensile expansion of cracks and microfractures up to a critical threshold where failure of the iceberg occurs through calving. The incidence of Type 1 and 2 events within the dominant 1–5 Hz frequency envelope possibly reflects continued cracking and damage accumulation produced by tensile stresses as the iceberg rolls to reach a new buoyant equilibrium following the loss of an ice block through calving (Supplementary Fig. 2). Thus, crack nucleation and expansion appear to progressively weaken icebergs in transit through the Vaigat Strait until mechanical stresses exceed the strength of the ice, culminating in a calving event.

### Magnitude-frequency scaling

**Figure 2:**
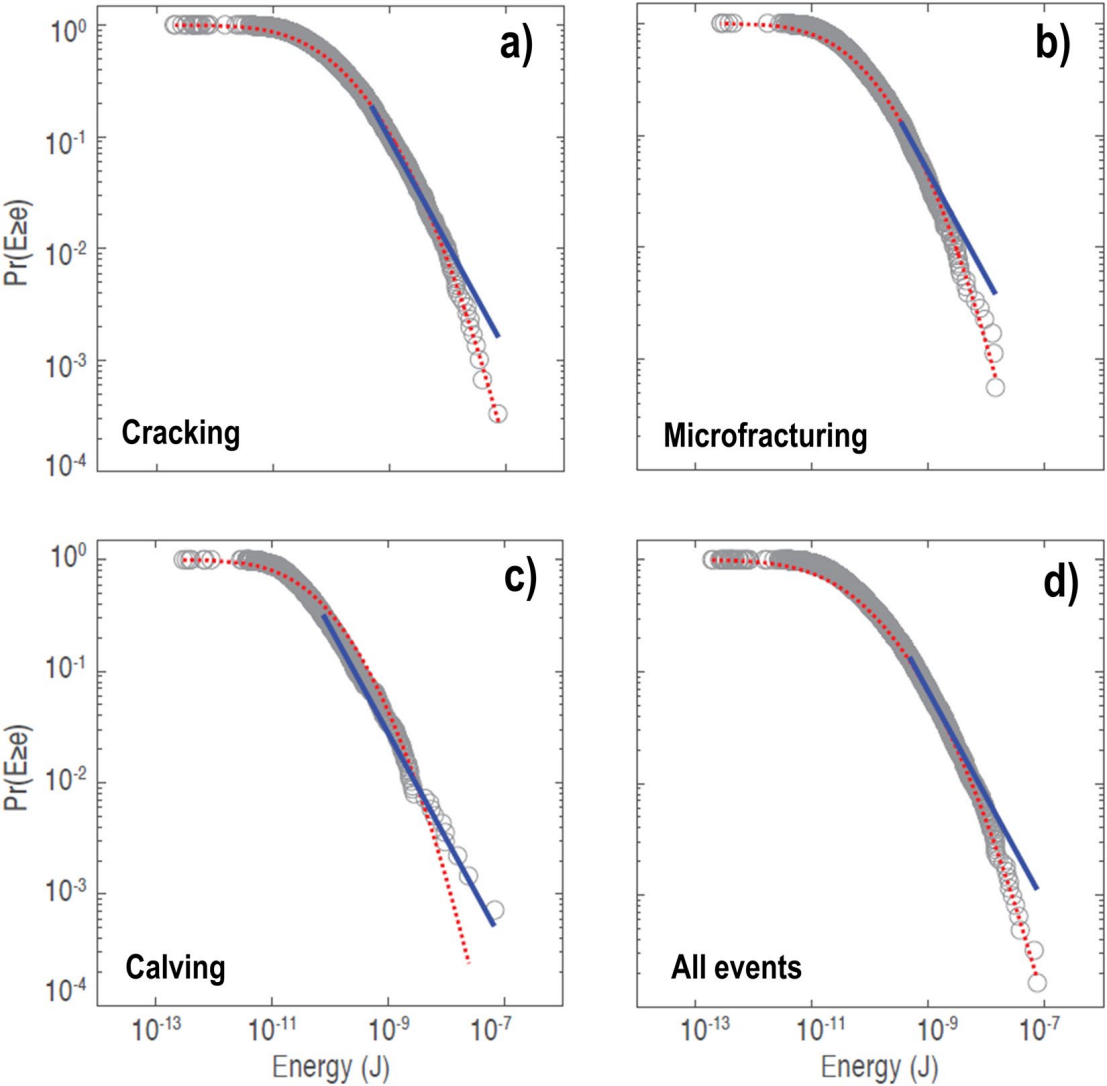
Cumulative size-frequency distributions, Pr(*E* ≥ *e*), for the energy released by iceberg fragmentation processes: (**a**) cracking, (**b**) microfracturing, (**c**) calving and rolling, (**d**) all detected events. Optimal lognormal and power-law approximations of the data are displayed as dashed red and solid blue lines, respectively.

Testing of different frequency distribution functions (outlined in Methods and Supplementary Methods 1) demonstrates that lognormal distributions provide the most robust analogue for the various iceberg disintegration processes. The spectrum of energies released by cracking and microfracturing are lognormally distributed over six orders of magnitude (Fig. 2a,b), with alternative power-law fits only providing a robust approximation of the data between signal energies of 5 × 10^−10^ J to 1 × 10^−8^ J and 3 × 10^−10^ J to 2 × 10^−9^ J, respectively. Power-laws thus overestimate the likelihood of occurrence for the smallest and highest magnitude cracking and microfracturing events — a pattern that is also observed for all events combined (Fig. 2d). A power-law approximation of the energy released by iceberg calving and rolling provides a robust fit to events with energies >8 × 10^−11^ J (n = 440) (Fig. 2c), but fails to predict the rollover of lower magnitude energies where the majority of the data (n = 961) falls. Despite overpredicting the likelihood of energies between 4 × 10^−11^ J and 1 × 10^−9^ J and underpredicting the incidence probability of the nine largest iceberg calving events, a lognormal distribution provides a better approximation of the data than the fitted power-law.

### Event timing

Event timing gives insight into the drivers of iceberg disintegration. Correlations between the number of icequakes detected and the height of the semi-diurnal tidal range are moderate to weak (r < 0.5) and vary considerably between the different processes detected (Supplementary Fig. 3a–c). However, greater numbers of events are observed across all six seismometers during periods coincident with the daily tidal range maxima, implying spatially consistent forcing driven by tides. The timing of iceberg calving, rolling and microfracturing exhibits comparable phasing to the lunar fortnightly (M_f_, 13.70 day) constituent tide, causing greater numbers of icebergs to calve, roll and fracture when transported into shallower coastal waters during periods of higher tidal amplitude, enabling keel grounding upon the seabed. These processes also exhibit a significant 2–4 day periodicity (Supplementary Fig. 3d) that may reflect progressive cycles of damage accumulation due to tidal grounding, culminating in iceberg disintegration after 2 to 4 days of repeated tensile loading, or amplified wave-notching as a result of increased ocean turbulence during the passage of transient storms^15^. Wave-driven turbulence disturbs the build-up of a static cold-water layer around icebergs that would diminish melt rates^40^, driving higher rates of heat transfer into the ice, increasing notch cutting and iceberg instability^1^. Similar periodic behaviour is not present for cracking-induced signals, which increase in prevalence throughout the study period (Supplementary Fig. 3a). This pattern likely relates to the cumulative expansion of cracks as a result of progressive microfracture nucleation and growth in response to storms through the summer season and iceberg grounding during periods of high tidal amplitude, permitting fractures to coalesce to produce the lower frequency signals associated with large-scale cracking.

## Discussion

**Figure 3:**
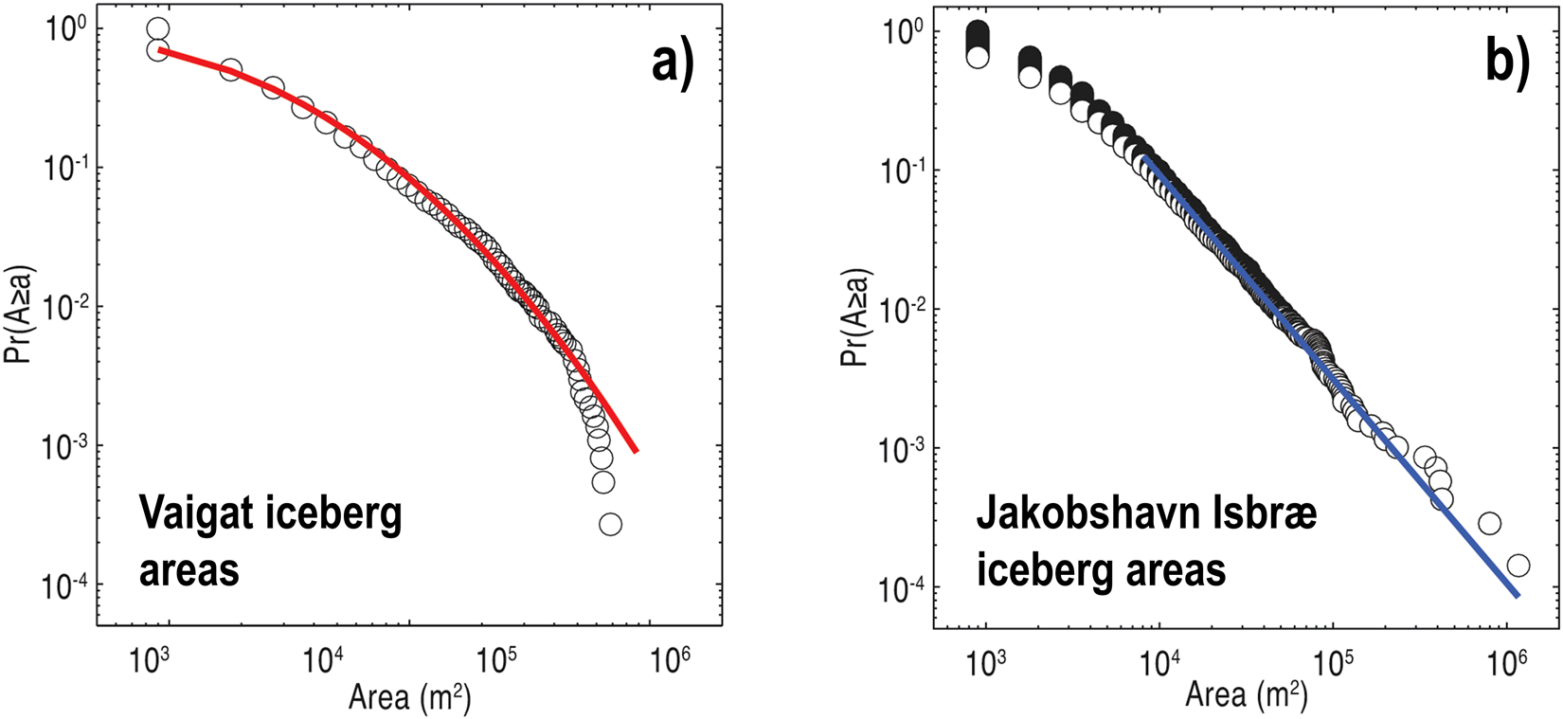
Optimal lognormal (red line) and power-law (blue line) approximations of the distribution of planform iceberg areas located: (**a**) within Vaigat and (**b**) proximal to Jakobshavn Isbræ. The slope of the fitted power law in (**b**) is 2.4.

Analysis of satellite imagery demonstrates that the distribution of planform iceberg areas in Vaigat is well fitted by a lognormal distribution except for the likelihood of the very largest icebergs, which are slightly overpredicted (Fig. 3a). The planform areas of icebergs situated in the zone proximal to the outlet of Jakobshavn Isbræ are power-law distributed over two orders of magnitude, with minor deviations from the fitted distribution occurring for icebergs with a planimetric area of less than 10,000 m^2^ (Fig. 3b). This result concurs with satellite-based analysis of the size-frequency distributions of icebergs located in other Greenlandic fjords situated within 200 km north of Jakobshavn Isbræ^41^, and within the Ilulissat Isfjord proximal to the calving terminus of Jakobshavn Isbræ^22^, suggesting that the size-frequency distributions of icebergs calved from Greenlandic outlet glaciers likely conform to power-law scaling. A power-law distribution of iceberg areas is consistent with observed and theoretical fragment-size distributions calved from tidewater glaciers and ice sheets^6^. The size-frequency distributions of iceberg sizes calved from glacial termini measured by seismic and imagery-based methods of monitoring are consistent regardless of the method used to conduct the monitoring (see supplementary methods in ref.^6^), indicating that: (i) seismic and satellite-derived measures of iceberg size-frequency distributions are compatible measures of iceberg size, and therefore that (ii) the process of iceberg calving operating at these glacial termini may be different from the lognormal distribution of energies associated with iceberg decay in the open waters of the Vaigat Strait.

The incidence of power-law scaling is indicative of a scale-invariant self-organised system fluctuating between regimes of sub-critical damage accumulation and super-critical instability collapse^6^. Systems exhibiting self-organised criticality evolve towards a critical state through the interaction of multiple simultaneous processes^42^. When the critical state is attained, accumulated instabilities may dynamically relax through scale-invariant avalanching, which in the context of a calving terminus may range from minor ice falls to the collapse of the entire calving front^6^. The progression to calving front instability is achieved through numerous mechanisms including surface ablation, longitudinal stretching, crevasse formation and submarine melt^43^. The connection of the structural damage generated by the various processes of instability propagation culminates in the mechanical failure of portions of the glacier terminus, producing icebergs at the calving margin^44^. Under these conditions, as no single mechanism of damage accretion dominates the iceberg calving process, the size-frequency distribution of the icebergs produced will not reflect a single formative process, resulting in the production of a scale-invariant power-law distribution of calved iceberg sizes^6^.

In contrast to the distributions generated at calving termini, power-law approximations of iceberg sizes observed across the North Atlantic Ocean over-predict both the smallest and largest iceberg dimensions^45^. The lognormal distribution of iceberg areas observed within Vaigat concurs with observations from the Arctic^7^ and Antarctic^8^, wherein distal icebergs obey lognormal scaling with minimal year-to-year variability^5^. The production of lognormal distributions has been theoretically^46^ and experimentally^47^ associated with multiplicative breakage and repeated fracturing. The theory of breakage represents an inverse application of the law of proportionate effect^48^ in which the value of a variable undergoing change corresponds to a random proportion of its previous value^49^. For a system governed by this law, assuming each transformation induced by breakage is small, application of the central limit theorem demonstrates that the logarithm of the variable undergoing change will be asymptotically normally distributed^50^, with the breadth of the lognormal distribution reflecting the number of independent transformations that are responsible for its formation^48,51^. The incidence of lognormal scaling in the distribution of energies released by iceberg fracturing suggests that the dominant mechanisms by which icebergs decay can be approximated as a process operating under the law of proportionate effect.

A lognormal distribution of iceberg sizes produced by fracturing processes is likely reinforced by the dimensional reduction of iceberg dimensions through melting. Although this process in itself has no detectable seismic signature, smaller icebergs generated through the fracturing process will exhibit greater surface area to volume ratios, making them more susceptible to mass loss through melting. The preferential removal of smaller icebergs from the total population through melting is conducive to the production of a rollover tail in the observed iceberg size-frequency distribution. The characteristic size-frequency distribution of iceberg fragments observed within the open ocean is therefore a function of the preferential loss of smaller icebergs through melting, facilitated by the tensile fracturing of larger icebergs.

Although initially unintuitive, the production of two different size-frequency distributions for icebergs, despite both being driven by fracture-dominated decay processes, may reflect the intrinsically connected nature of lognormal and power-law distributions as demonstrated by the fact that both may be produced using similar basic generative models^51^. The distributional breadth of lognormal distributions increases as the active processes responsible for their generation become more intricate and numerous^52^. As the complexity, and thus breadth, of the distribution increases, lognormal distributions begin to exhibit properties that are more commonly associated with power-law behaviour, providing a greater extent of overlap in which these two distributions are indistinguishable^53^ (Fig. 1c).

Reversing this logic, power-law distributed phenomena exposed to a breakage process tend towards lognormality as the complexity of the degradation mechanisms reduces owing to the emergence of a prevailing process subset. The dominance of three iceberg-fragmentation mechanisms within Vaigat, compared to the multitude of damage accretion mechanisms operating at glacial termini, suggests that the number of processes driving iceberg disintegration reduces as icebergs drift away from the calving front. It is this simplifying phenomenon that drives the transition from a power-law distribution at and proximal to a calving front, to the lognormal distribution of iceberg sizes observed beyond. As the breadth of a lognormal distribution decreases as the number of processes responsible for its formation reduces^51^, the size-frequency characteristics of iceberg populations will become increasingly lognormal as the mechanisms of iceberg decay continue simplify with further distance away from the calving front (Fig. 1c). We therefore anticipate that upon successful transport through Vaigat to deeper open waters, the absence of tidal grounding will further promote the dominance of a smaller number of wave and melt-based processes, consequently reinforcing the lognormality of the observed iceberg distribution.

The largest icebergs calved from Jakobshavn Isbræ are commonly over 1000 m in length, several hundred metres wide and exhibit keel depths of up to 900 m^37^. However, the seaward transportation of the largest icebergs into Disko Bay is impeded by the relatively shallow water of the Isfjeldsbanken bank, stranding those with draughts >200 m until a sufficient reduction in size occurs through fragmentation and/or melt^4^. Consequently, icebergs in the heavy tail of the power-law distribution proximal to Jakobshavn Isbræ are left stranded, which may explain why the lognormal approximation of iceberg areas within Vaigat marginally overestimates the likelihood of the largest icebergs (Fig. 3a). The transition from a power-law to a lognormal distribution of iceberg sizes can therefore begin in coastal waters, here close to the calving margin at Isfjeldsbanken, although localised differences in coastal bathymetry will constrain the distance away from the ice margin that this transition will initiate for other calving fronts.

The exclusion of the largest icebergs gives an example of a shift in the mechanisms of iceberg decay operating within and beyond Disko Bay. Flexure of icebergs by waves can cause larger icebergs to fatigue, fracturing along pre-existing flaws^40^. However, for icebergs <1,000 m in length, the impact of this process becomes negligible, leaving mass loss to be dominated by wave-related mechanisms such as the collapse of wavecut overhangs, buoyant failure of protruding underwater rams and forced thermodynamic convection due to differential iceberg-water velocities^1^. As the shallow water at Isfjeldsbanken prevents the very largest icebergs being transported beyond Disko Bay, the complexity of iceberg-fragmentation processes operating within these waters is significantly reduced in comparison to those present at the calving front, permitting a small number of decay mechanisms to dominate. Thus, whilst calving may generate power-law frequency distributions of iceberg size, those leaving coastal waters may more likely adhere to lognormal size distributions.

Passive seismic monitoring therefore suggests that, owing to the fracture-driven iceberg disintegration processes and dimensional reductions through melting, iceberg size-frequency distributions will exhibit an increasingly definitive lognormal shift with drift away from the calving front. This shift can be explained by the emergence of a dominant set of driving processes of iceberg degradation as icebergs transit towards the open ocean. Although lognormal and power-law distributions both provide credible models for the mid-range values of many empirical data, adequately representing the tail of a distribution has significant consequences for predicting the future behaviour of a phenomenon^51^. Whilst the heavy-tailed nature of power laws is required to model icebergs in regions close to calving fronts, the use of a power-law distribution to estimate the occurrence probability of seaward icebergs overpredicts the numbers of the largest and smallest iceberg dimensions. A lognormal alternative is therefore needed to model iceberg distributions accurately, and from this to derive risk and rates of iceberg disintegration.

## Methods

### Data collection and processing

Seismic signals generated by the processes of iceberg decay were recorded over a 49-day period between 18^th^ July and 4^th^ September 2013 using six Güralp ESPCD broadband seismometers installed in coastal locations along a 50 km stretch of the Vaigat Strait. The vertical component of ground motion was detrended before being filtered using a 1–50 Hz Butterworth band-pass filter to attenuate noise generated by ocean waves and distal earthquakes. Events were detected using the ratio of the root mean square of short-term moving average (2 s) and long-term moving average (60 s) windows, with events being retained for further analysis when the ratio exceeded a threshold of 10. Cross-correlation of signal arrival times for each seismometer revealed that the detected events are highly localised and generally only exceed the retention threshold at a single station. Arrival time differences between the P-wave and S-wave component of signals demonstrates that the source of the detected events is located within ~15 km of the associated seismometer (Fig. 1). The detected signals are therefore sourced from processes operating within the Vaigat Strait and are not duplicated across multiple seismometers.

### Event classification

Previous investigations of glaciological phenomena using passive seismic techniques have demonstrated that different glacial processes are characterised by unique and highly distinctive signal properties including dominant spectral frequency, event duration and the shape of the signal onset and coda (Table 1). On detection, the characteristic frequency of individual signals was examined using a combination of spectrograms and power spectral density estimations. Signal duration and the profile of each signal onset and coda were manually described on the basis of visual inspection. By using a threshold of signal power relative to the background noise in each detection envelope, it was possible to describe each detected signal in terms of an envelope dominant frequency and duration, whilst the shape of the onset and coda of each signal was classified as either impulsive or emergent. Using characteristic spectral frequency, duration and the shape of the signal onset and coda as distinguishing properties, the 6842 events detected by the seismometer array were grouped into three signal categories (Supplementary Fig. 1; Table 2). Dominant spectral frequency was the clearest descriptor of signal type. However, the detected events appear to be drawn from a continuum of processes; hence distinctions between signal types were often ambiguous, with some events consisting of a sequence of all three types of events combined (Supplementary Fig. 2).

### Magnitude-frequency analysis

The energy released by each detected signal was calculated using methods introduced by Amitrano *et al*.^54^ in a study of cliff collapse in Normandy, France. Following the grouping of all events into process-related classifications, the complementary cumulative size-frequency distribution (CSFD), of signal energies was used to assess the size-frequency characteristics of each iceberg disintegration process. The CSFD denotes the probability Pr(*E* ≥ *e*) that the energy of an event, *E* (J), exceeds a given energy, *e* (J)^30^. Technical assessment^55^ of the cumulative size-frequency distributions generated by each iceberg disintegration mechanism indicated that both power-law and lognormal distributions could provide potential models for the iceberg disintegration process. In order to establish which of these competing distributions provided the most credible model for the data, best-fit parameters for each distribution, including the minimum boundary for which the model applies (x_min_), were derived using maximum likelihood estimation^56^. Directly competing power-law and lognormal models for the data were then compared using Vuong’s test^57^ — a likelihood-ratio test using the Kullback-Leiber criterion^58^. The sign of the likelihood ratio, *R*, indicates which distributional model provides the best fit to the data^56^. Here, *R* is positive if the power-law model provides the better fit, negative if a lognormal model provides the best fit, and zero if the fit provided by a distribution is indistinguishable from its alternative. The statistical significance of the sign of *R* is given by a *p*-value. If *p* is small (*p* < 0.1), it is unlikely that the observed sign of *R* may vary due to statistical fluctuations and thus may be used to comment on which distribution provides the most robust fit^56^. This analysis was conducted using the poweRlaw package in the statistical software R^58,59^. This analysis is presented in further detail in Supplementary Methods 1.

### Comparison with satellite imagery

Icebergs in transit through the Vaigat Strait predominantly originate from Jakobshavn Isbræ. A comparison between the dimensions of icebergs present within the Vaigat Strait compared to those located proximal to Jakobshavn Isbræ was derived from a 1500 km^2^ contemporaneous Landsat 8 image of Vaigat (image ID: LC80110112013259LGN00, 09.16.2013) and a 1800 km^2^ Landsat 7 image of Jakobshavn Isbræ (image ID: LE70100112013196EDC00, 06.15.2013). Both images share the same 30 m resolution, permitting a direct comparison to be made between the areal properties of the icebergs present in each area. Iceberg areas were delineated using an automated algorithm based on the contrast between the icebergs and the surrounding seawater. In order to ensure that the iceberg dimensions were accurately delineated, the contrast between the icebergs and the surrounding seawater was first increased using a global image threshold based on Otsu’s method. The validity of the mapping algorithm was then manually checked to ensure that closely grouped patches of icebergs were not interpreted as one large ice mass; any areas in which this issue was present were excluded from the analysis. The magnitude-frequency characteristics of the extracted iceberg area populations were then analysed in the same manner as the seismic signal energies, detailed in Methods: Magnitude-frequency analysis.

### Correlation with tides

Modelled hourly tide data for Ilulissat (~120 km from Vaigat) between the 18^th^ of July and 30^th^ of August was provided by the Danish Meteorological Institute, Copenhagen. A time-lag correction of 2 hours relative to Ilulissat was applied in order to make the data applicable to Vaigat, based upon analysis of the tidal signal in the seismic data^60^. The timing of detected icequakes was compared against periodic components of the modelled tidal cycle in order to examine any potential relationship between tidal forcing and seismic signal incidence. As the uneven time interval between observations inhibits the application of typical fast Fourier transform techniques to assess the periodicity of icequake signals, the Lomb-Scargle periodogram, which is designed to examine unevenly spaced time series^61^, was used to estimate the power spectrum of the icequake time series, binned into 6-hour intervals. Bin width had a negligible effect on the calculated spectral power.

### Data availability

The seismic data used in this study are available from the IRIS MDC data repository (http://ds.iris.edu/ds/nodes/dmc/data/), initially on request of the corresponding author. Following publication, the data will be made fully open-access after 2–3 years, in line with SEIS-UK policy on data availability^62^.

## Electronic supplementary material

Supplementary methods
