# Supplementary material for: Drift-dependent changes in iceberg size-frequency distributions
Source: Sci Rep. 2017 Nov 22;7:15991. doi: 10.1038/s41598-017-14863-2 (PMC5700179; doi:10.1038/s41598-017-14863-2)
Supplement: Supplementary file 1 — Supplementary methods [file 41598_2017_14863_MOESM1_ESM.doc]

**Supplementary methods for: Drift-dependent changes in iceberg size‑frequency distributions**

James D. Kirkham^1,2^**^*^**

Nick J. Rosser^1^

John Wainwright^1^

Emma C. Vann Jones (née Norman)^1^

Stuart A. Dunning^3^

Victoria S. Lane^4^

David E. Hawthorn^5^

Mateusz C. Strzelecki^6^

Witold Szczuciński^7^

^1^Geography Department and Institute of Hazard Risk and Resilience, Durham University, Durham, DH1 3LE, UK. ^2^Scott Polar Research Institute, University of Cambridge, Cambridge, CB2 1ER, UK. ^3^School of Geography, Politics and Sociology, Newcastle University, Newcastle, NE1 7RU. ^4^SEIS-UK, Department of Geology, University of Leicester, Leicester, LE1 7RH, UK. ^5^British Geological Society, The Lyell Centre, Edinburgh, EH14 4AP, UK. ^6^Institute of Geography and Regional Development, University of Wrocław, 50-137 Wrocław, Poland. ^7^Institute of Geology, Adam Mickiewicz University in Poznań, 61-680 Poznań, Poland.

^*^email: jk675@cam.ac.uk

1. **Additional notes on distribution testing using Vuong’s test**

In order to conduct Vuong’s test^29^, both model distributions must have the same minimum boundary threshold (x_min_). As the value of x_min_ varied between the two potential distributional fits, Vuong’s test was repeated using the x_min_ values of both distributional candidates in order to gauge which distribution produced the best fit to the data (Supplementary Table 1). Lognormal models of each process’ energy distribution produced x_min_ values up to two orders of magnitude lower than their power-law counterparts, providing a fit over a greater range of the data. The sign of *R* for all likelihood ratios is negative, indicating that the lognormal models provide the most adequate fit for each distribution. When the power-law x_min_ threshold is used to conduct Vuong’s test, this result is statistically significant (*p* < 0.1) for the process of cracking and for all events combined, however the difference between power-law and lognormal models for microfracturing and calving is insufficient to be statistically distinguishable. However, when a larger proportion of the data is examined using the lower x_min_ threshold estimated for lognormal models, all tests indicate a statistically significant difference between power-law and lognormal models for the data, with lognormal estimations providing the better fit. As lognormals provide a better model for the event energy distributions than power-laws over a wider proportion of the data, we conclude that lognormal distributions provide the most robust analogue for the various iceberg disintegration processes.

**References of the main text**

1. Vuong, Q.H. Likelihood ratio tests for model selection and non-nested hypotheses. *Econometrica* **57**, 307-333 (1989).

**Supplementary Figures**

**Supplementary Figure 1 | Characteristic frequencies and durations of the three types of signals observed.** a) Dominant spectral frequency of the events detected by the seismogram array. Although some overlap is present, three dominant spectral frequency peaks are observed between 2 – 8 Hz, 8 – 30 Hz, and 25 – 45 Hz. b) Duration of the detected seismic signatures classified by dominant spectral frequency. Types 1 and 2 typically last for < 4 seconds, but are observed to range between ~ 0 and 15 seconds in duration, whilst Type 3 events are associated with a longer characteristic duration of 2 – 20 seconds. Type 1, 2 and 3 signals are interpreted as microfracturing, tensile crack enlargement and iceberg calving and rolling, respectively.


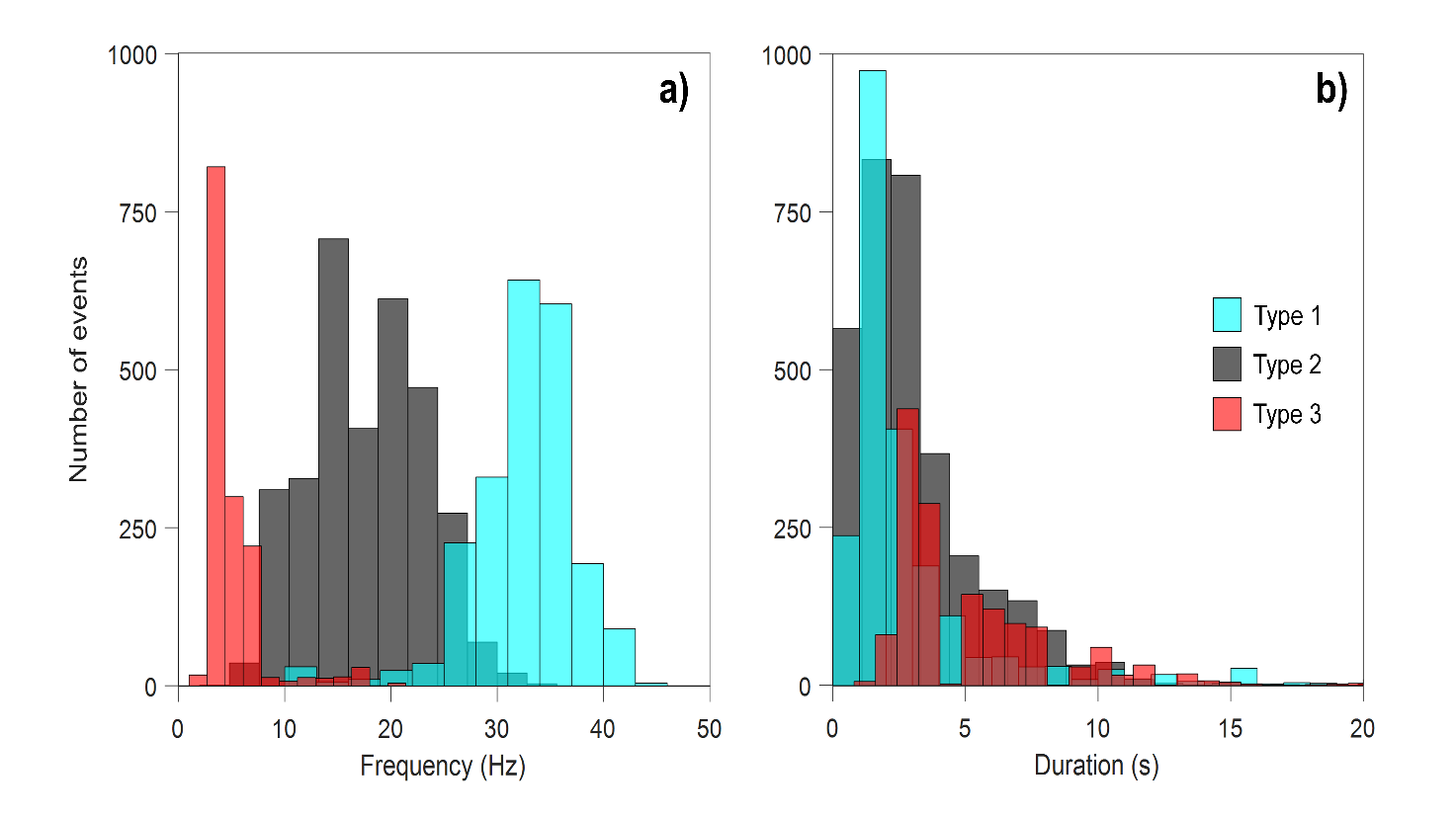


**Supplementary Figure 2 | The continuum of iceberg-related processes operating in the Vaigat Strait.** a) Schematic illustration of the processes responsible for generating the seismic signals detected. Type 1 microfractures expand the tips of pre-existing cracks leading to large scale crack enlargement (Type 2). When the stress imposed on the iceberg by a crack exceeds the strength of the remaining ice, a block of ice is calved from the iceberg (Type 3) resulting in rolling and tilting as the iceberg readjusts to a new buoyant equilibrium. b) Waveform generated by a typical calving event. c) Spectrogram of a typical calving event displaying the continuum of processes which lead to iceberg disintegration: (1) low-power microfracturing leads to the expansion of a larger crack (2), leading to the calving of a block of ice which impacts with the water surface and causes the iceberg to roll (3). A series of high frequency (20 – 40 Hz) peaks coincides with the dominant 1 – 10 Hz frequency associated with calving and rolling, suggesting that the motion of the iceberg during restabilisation instigates further expansion and nucleation of cracks.


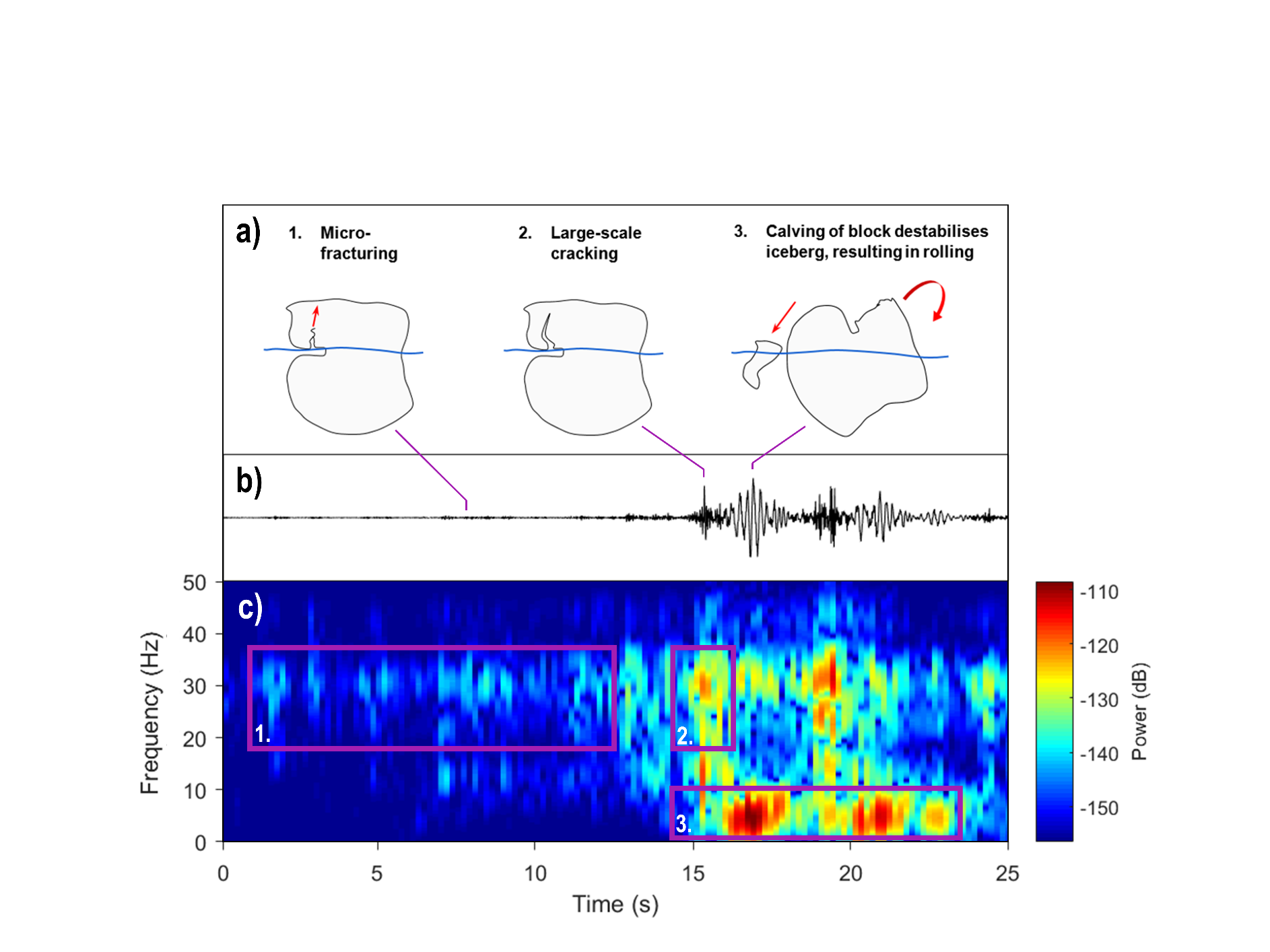


**Supplementary Figure 3 | Periodicities of icequake signals.** (a-c) Comparison between the number of events detected per day (bars) and the height of the higher-high to lower-low tidal range (dashed line) for a) cracking, b) microfracturing and c) calving events. r values denote the correlation between tidal range and the number of events detected per day. d) Lomb-Scargle periodogram displaying the power spectrum of the Vaigat icequake time series for all detected events, binned into 6-hour intervals. Dashed lines correspond to false alarm probabilities, α, of 50 % and 5 %, denoting the probability that a peak with a power exceeding this threshold is the result of random statistical fluctuations alone. The period, in days (d), of several prominent peaks is labelled.


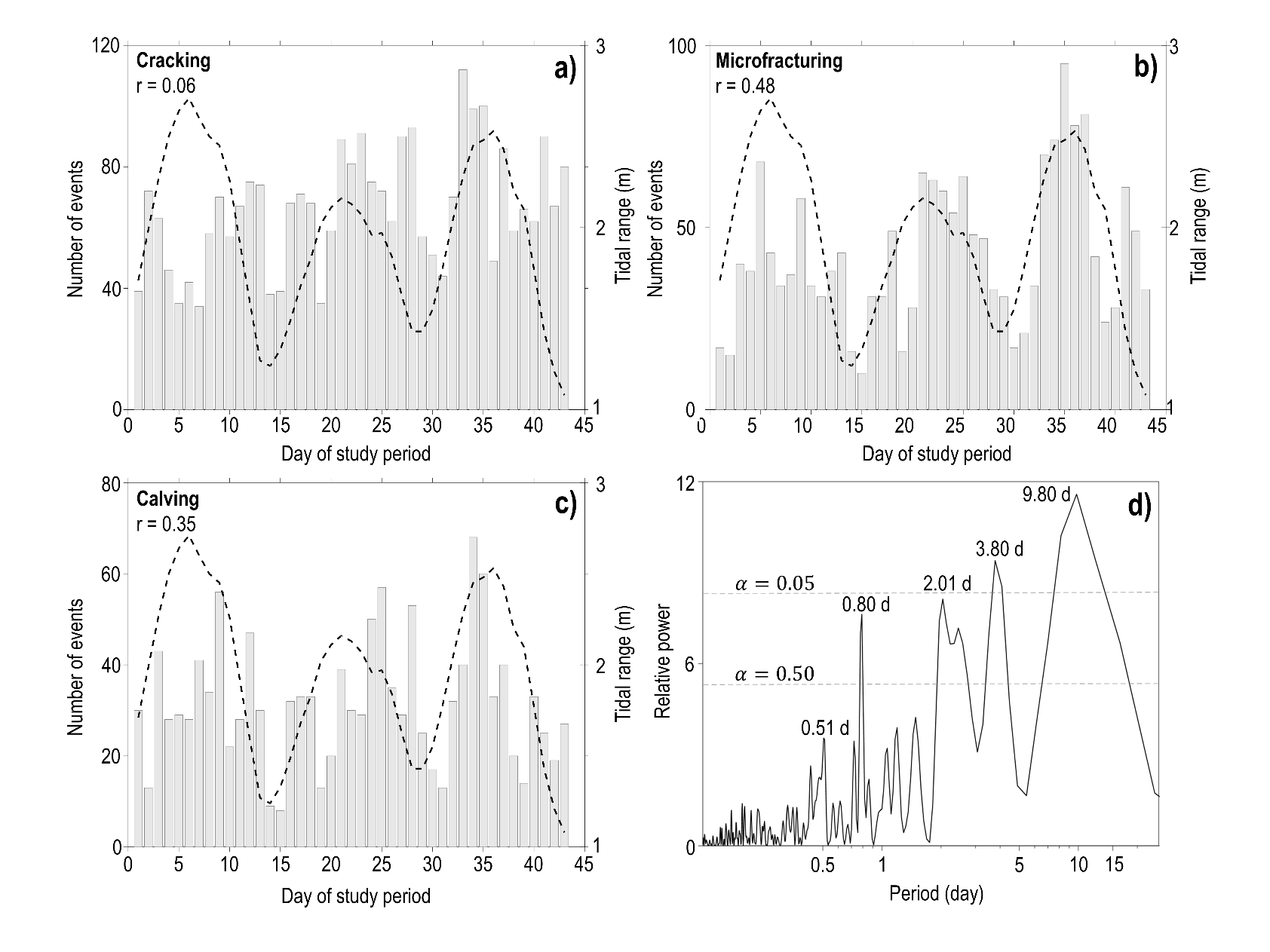


**Supplementary Tables**

| **Supplementary Table 1 | Model parameters and goodness-of-fit test results for the energy distributions of events detected by the seismometer array.** | | | | | | | | | | | | |
| --- | --- | --- | --- | --- | --- | --- | --- | --- | --- | --- | --- | --- |
| **Process** | **Power-law α** | **Power-law x_min_ (J)** | | | **Lognormal µ** | **Lognormal σ** | | **Lognormal x_min_ (J)** | **R (power-law x_min_)** | ***p*-value** | **R (lognormal x_min_)** | ***p-*value** |
| Cracking | 1.95 | 4.86 x 10^-10^ | | | -23.1 | 1.81 | | 6.21 x 10^-12^ | -2.01 | 0.04 | -24.4 | 2.66 x 10^-131^ |
| Microfracturing | 2.20 | 3.38 x 10^-10^ | | | -23.7 | 1.61 | | 6.02 x 10^-12^ | -0.79 | 0.43 | -12.0 | 3.42 x 10^-33^ |
| Calving | 1.86 | 7.50 x 10^-11^ | | | -24.1 | 1.64 | | 4.86 x 10^-12^ | -1.05 | 0.29 | -7.77 | 7.74 x 10^-15^ |
| All | 1.99 | 4.00 x 10^-10^ | | | -23.6 | 1.81 | | 6.09 x 10^-12^ | -2.69 | 0.01 | -23.8 | 2.40 x 10^-125^ |
|  | | | | | | | | | | | | |
|  | | |  |  | | |  | | | | | |
